# Bufalin suppresses hepatocarcinogenesis by targeting β-catenin/TCF signaling via cell cycle-related kinase

**DOI:** 10.1038/s41598-018-22113-2

**Published:** 2018-03-01

**Authors:** Zhuo Yu, Hai Feng, Xuehua Sun, Yunhui Zhuo, Man Li, Zhenhua Zhou, Lingying Huang, Yun Jiang, Xiaojun Zhu, Xin Zhang, Fan Le, Chao Zheng, Alfred Szelok Cheng, Yueqiu Gao

**Affiliations:** 1 Liver Disease Department, Shuguang Hospital Affiliated to Shanghai University of Traditional Chinese Medicine, Shanghai, P.R. China; 2 Laboratory of Cellular Immunity, Shuguang Hospital Affiliated to Shanghai University of Traditional Chinese Medicine, Shanghai, P.R. China; 3 Department of pharmacology, School of Pharmacy, Harbin Medical University, Harbin, P.R. China; 4 School of Biomedical Sciences, State Key Laboratory of Digestive Disease, The Chinese University of Hong Kong, Hong Kong, SAR P.R. China

## Abstract

Hepatocellular carcinoma (HCC) is one of the most aggressive malignant tumors, of which treatment options are limited especially in advanced stage. Bufalin, the major digoxin-like component of the traditional Chinese medicine Chansu, exhibits significant antitumor activities in hepatoma cells, but the potential mechanism is obscure. Cell cycle-related kinase (CCRK) is recently identified to be a crucial oncogenic master regulator to drive hepatocarcinogenesis. Here we investigated the molecular function of bufalin on CCRK-regulated signaling pathway, and expounded the underlying mechanism in HCC suppression. *In vitro* with PLC5 HCC cells and human immortal LO2 cells, proliferation, malignant transformation and cell cycle progression assays were performed to evaluate the antitumor effect of bufalin. *In vivo* with xenograft and orthotopic mice models, tumor growths with weight and volume change were assessed with or without bufalin treatment. Western blot, RT-qPCR, immunofluorescence and immunohistochemistry were conducted to examine the expression level of CCRK and β-catenin/TCF signaling cascade. We revealed that bufalin suppresses PLC5 HCC cell proliferation, transformation and cell cycle progression rather than LO2 cells, which is correlated with CCRK-mediated β-catenin/TCF signaling. It was also confirmed in mice model. Thus, bufalin is a potential anti-HCC therapeutic candidate through the inhibition of CCRK-driven β-catenin/TCF oncogenic signaling pathway.

## Introduction

Hepatocellular carcinoma (HCC) is one of the most malignant neoplasms with 750,000 deaths each year, seriously threatening human health worldwide^1^. Surgical resection, liver transplantation and radiofrequency ablation are the preferred therapeutic strategies in the treatment of HCC^2,3^. However, only 20% of 5-year survival rate post operation for HCC patients greatly decreases surgical therapeutic effect and the recurrence is still increasing due to malignant invasion and metastasis of tumor cells^4,5^. In addition, the feature of HCC to be resistant to chemotherapeutic cytotoxicity restricts the application of the conventional chemotherapeutic agents for the treatment of HCC^6,7^. The multikinase inhibitor sorafenib improves clinical benefit of HCC treatment by targeting cell proliferation-related signaling pathways involved in genetic regulation and epigenetic modification^8,9^, shedding light on the development of novel therapeutic strategies in HCC distinct from conventional therapeutic medicines. Therefore, identification of novel unconventional chemotherapeutic medicines and exploration of brand-new underlying mechanisms are still urgent for improving efficacy of HCC treatment.

Traditional Chinese medicine (TCM) cinobufacini, which is extracted from the skins and parotid venom glands of *Bufo bufo gargarizans* Cantor, has been shown to have potent antitumor activities in several clinical trials and has attracted increasing interests as a promising candidate for developing novel therapeutic regimens in cancer^10–12^. Bufalin is one of the major active ingredients of cinobufacini with the potential effect on inhibiting numerous neoplastic developments including HCC^12,13^. It has been reported that bufalin suppresses invasion and metastasis of hepatoma cells by regulating multiple proliferation-related signaling pathways such as PI3K/AKT/mTOR signaling and NF-κB/matrix metalloproteinase-2/-9 signaling^14,15^. Other recent studies have shown that bufalin strengthens the ability of sorafenib to suppress HCC proliferation through a synergistic effect^16,17^. These findings indicate a distinct mechanism underlying bufalin-induced HCC suppression differing from the cytotoxic effect of conventional chemotherapeutic drugs, which needs to be further investigated.

The functional disorder of β-catenin/TCF signaling makes a great contribution to the neoplastic proliferation and transformation in most HCCs^18^. Besides genetic mutation, the aberrant activation of β-catenin induced by various modulators such as IL-6 promotes hepatocellular tumorigenicity by enhancing its carcinogenesis potential^19^. Cell cycle-related kinase (CCRK) is a cell cycle regulator that mediates the effect of cell growth in vital physiological and pathological process, including cancer initiation and progression^20,21^. In HCC, we found that CCRK functions as an oncogenic master modulator to induce activation and nuclear translocation of β-catenin, where it forms a complex with nuclear transcription factor TCF. The complex binds to its target specific DNA sequence in the nuclei, leading to the upregulation of several pro-proliferative factors such as cyclin D1 (CCND1) and epidermal growth factor receptor (EGFR)^21,22^. Further functional analysis confirmed that CCRK drives β-catenin/TCF-dependent hepatocarcinogenesis via dysregulating cell cycle progression^23,24^. These results consolidate that CCRK is a potential target for developing new therapeutic regimen against HCC.

Bufalin has been reported to interfere with β-catenin activity and cell cycle progression, however, the exact influence of bufalin on CCRK in suppressing hepatic neoplasm is not fully understood. In the current study, we investigated the role of bufalin in CCRK-induced hepatocarcinogenesis by functional analysis associated with gene expression. It was shown that bufalin directly inhibits CCRK expression in HCC cells, giving rise to G1 phase arrest in cell cycle. *In vitro* and *in vivo* experiments, we further disclosed that bufalin suppresses *CCRK* transcription by reducing the binding ability and transcriptional activity of *CCRK* promoter, thereby inactivating β-catenin/TCF pathway to suppress HCC cell proliferation and tumorigenicity.

## Results

### Bufalin suppresses HCC cell proliferation, transformation and cell cycle progression

**Figure 1:**
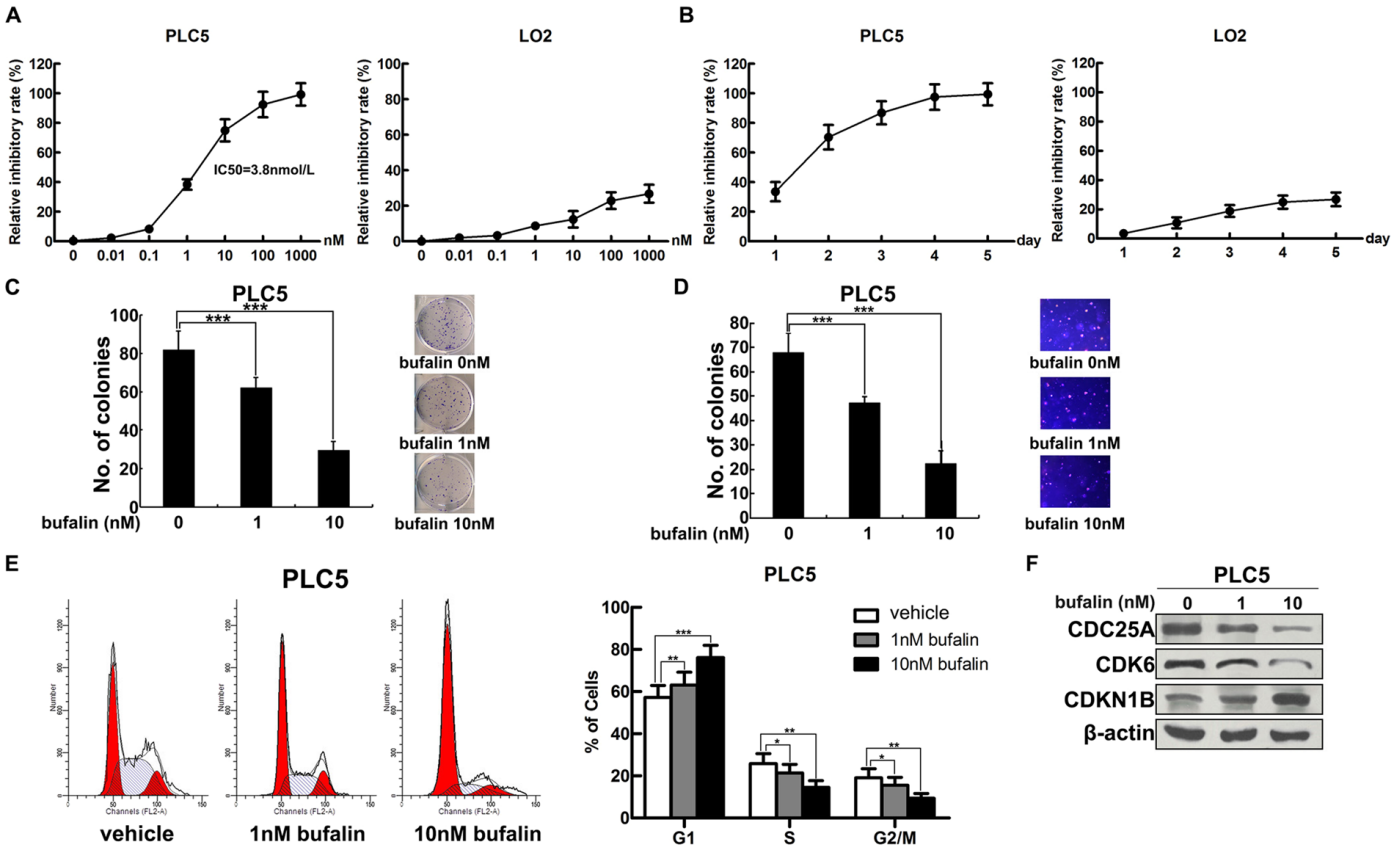
Bufalin induces G1 phase arrest to inhibit HCC cell proliferation and transformation. (**A**) Bufalin inhibited the proliferation of PLC5 cells in a dose-dependent manner compared with LO2 cells. Indicated concentrations of bufalin were treated with PLC5 or LO2 cells for 48 hours. Cell viability was measured in CCK-8 assay and the inhibitory rate was calculated referring to vehicle treatment. (**B**) Bufalin inhibited the proliferation of PLC5 cells in a time-dependent manner compared with LO2 cells. 10 nmol/L of bufalin was incubated with PLC5 or LO2 cells for 5 consecutive days. Cell viability was measured every 24 hours and the inhibitory rate was calculated referring to vehicle treatment. (**C**) Bufalin inhibited focus formation of PLC5 cells in a dose-dependent manner. Representative images of colonies formed are shown. (**D**) Bufalin inhibited anchorage-independent growth of PLC5 cells in soft agar. Representative images of colonies formed are shown. Original magnification, ×100. (**E**) Bufalin impeded cell cycle transition by inducing G1 phase arrest in PLC5 cells. (**F**) The protein levels of CDC25A, CDK6 and CDKN1B were detected by western blot in bufalin-treated PLC5 cells. β-actin was used as an loading control. *P < 0.05; **P < 0.01; ***P < 0.001.

To explore the effect of bufalin on the growth of hepatic carcinoma cells, PLC5 HCC cells comparing with human immortalized LO2 hepatocytes were treated with bufalin at 7 different doses ranging from 0 to 1000 nmol/L. After 48 hours incubation, cell viability was measured by CCK-8 assay. In contrast with less influence on the growth of LO2 cells, bufalin exhibited strong ability to suppress the number of PLC5 cells in a dose-dependent manner (Fig. 1A). The IC_50_ of bufalin on PLC5 cells is 3.8 nmol/L, while this dose only inhibited less than 10% growth of LO2 cells. Even up to the dose of 1000 nmol/L, which almost eliminated all PLC5 cells, merely reduced 20% of LO2 cells. In addition, we individually incubated 10 nmol/L of bufalin with PLC5 or LO2 cells for consecutive 5 days and examined cell viability at indicated time points. Bufalin prominently showed time-dependent manner in suppressing PLC5 cell growth, but limited effect in LO2 cells (Fig. 1B). For 24 hours incubation, bufalin achieved 30% inhibition rate on PLC5 cells in contrast to no inhibition in LO2 cells. With the incubation time increased to 96 hours, the inhibition rate in PLC5 cells was close to 100%, while merely 20% in LO2 cells. These data indicated higher suppression efficacy of bufalin on HCC cells than normal hepatocytes. We further assessed the impact of bufalin on the malignant proliferation and transformation of HCC cells by performing colony formation and soft agar assay. 1 nmol/L and 10 nmol/L of bufalin were separately incubated with PLC5 cells and each dosage markedly reduced focus formation (*P* < 0.001; Fig. 1C) and anchorage-independent growth (*P* < 0.001; Fig. 1D) compared with vehicle treatment. These results strengthen the ability of bufalin inhibiting the malignant proliferation and transformation of PLC5 HCC cells in a dose-dependent manner. In cell cycle analysis, PLC5 cells were treated with different doses of bufalin or vehicle for 4 hours and then detected by propidium iodide-staining flow cytometry. Bufalin significantly impeded G1/S phase transition by holding cells back in G1 phase, while more cells entering S and G2/M phase in control group (*P* < 0.001; Fig. 1E). This finding was further consolidated by the decreased expression of CDC25A and CDK6, the checkpoint of G1/S phase transition, and the increased expression of p27KIP1 (CDKN1B) which represents G1 phase arrest in bufalin-treated cells compared to controls (Fig. 1F). Moreover, apoptosis-related protein expressions of active caspase3 and cleaved PARP were undetected after bufalin treatment (data not shown). Taken together, bufalin effectively prefers to inhibit hepatoma cell proliferation rather than hepatic cell.

### Bufalin inactivates β-catenin/TCF signaling in HCC cells

**Figure 2:**
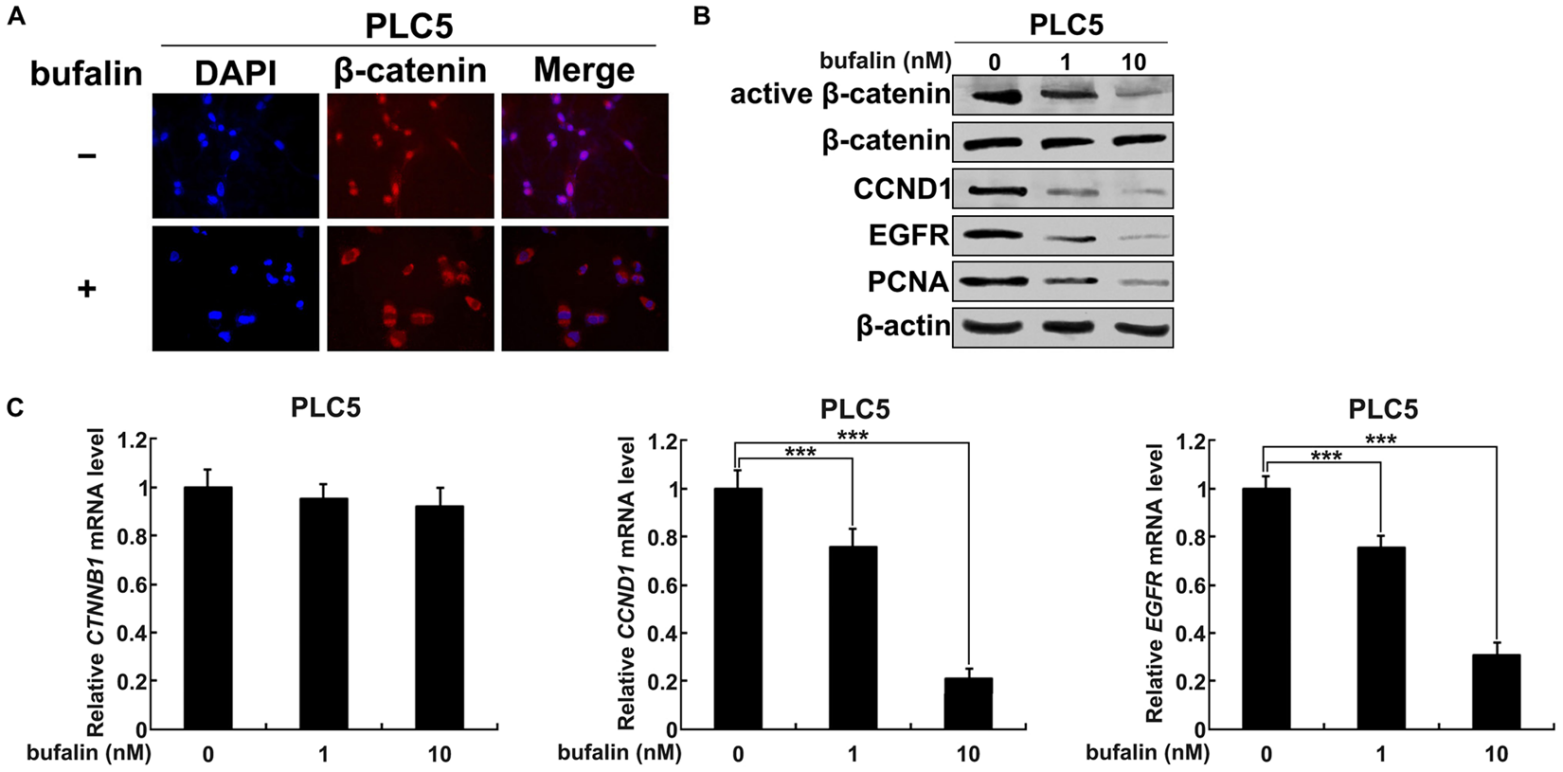
Bufalin inhibits β-catenin/TCF signaling in HCC cells. (**A**) Bufalin induced the redistribution of β-catenin from nuclei to cytoplasm. Red immunofluorescence staining of β-catenin was shown in representative images and nuclei were counterstained with DAPI. (**B**) Bufalin inactivated β-catenin signaling in a dose-dependent manner. Western blot analysis was performed to detect the expression of active and total β-catenin, CCND1, EGFR and PCNA. β-actin was used as a loading control. (**C**) Bufalin decreased *CCND1* and *EGFR* mRNA levels, but not *CTNNB1*, in a dose-dependent manner. Various concentrations of bufalin were incubated with PLC5 cells and quantitative RT-PCR was performed to detect transcript levels. ***P < 0.001.

Given the crucial role of β-catenin/TCF signaling in promoting HCC development through the regulation of cell cycle^25,26^, we investigated the impact of bufalin on β-catenin activity in suppressing HCC proliferation. In HCCs, β-catenin in the cytoplasm is mostly activated (dephosphorylated) and transfers into the nuclei, where it physically interact with TCF transcription factors to regulate transcription^27,28^. To localize β-catenin in PLC5 cells, immunofluorescence staining was performed following the treatment with 10 nmol/L of bufalin and vehicle respectively. In contrast to nuclear accumulation of β-catenin in vehicle group, bufalin led to redistribution of β-catenin to cytoplasm (Fig. 2A). Western blot analysis further confirmed that bufalin decreased the level of active β-catenin, but not total β-catenin (Fig. 2B). Consistently, β-catenin inactivation reduced the expression of downstream pro-proliferative targets, CCND1 and EGFR, leading to the abrogation of HCC proliferation evidenced by the inhibition of cellular proliferation marker PCNA (Fig. 2B). Quantitative RT-PCR further confirmed that bufalin was unable to affect gene transcription of *CTNNB1*, but dose-dependently decreased the mRNA levels of *CCND1* and *EGFR* (Fig. 2C). These results demonstrate that inactivation of β-catenin/TCF signaling is involved in bufalin-suppressed HCC proliferation.

### Bufalin decreases CCRK expression in HCC cells

**Figure 3:**
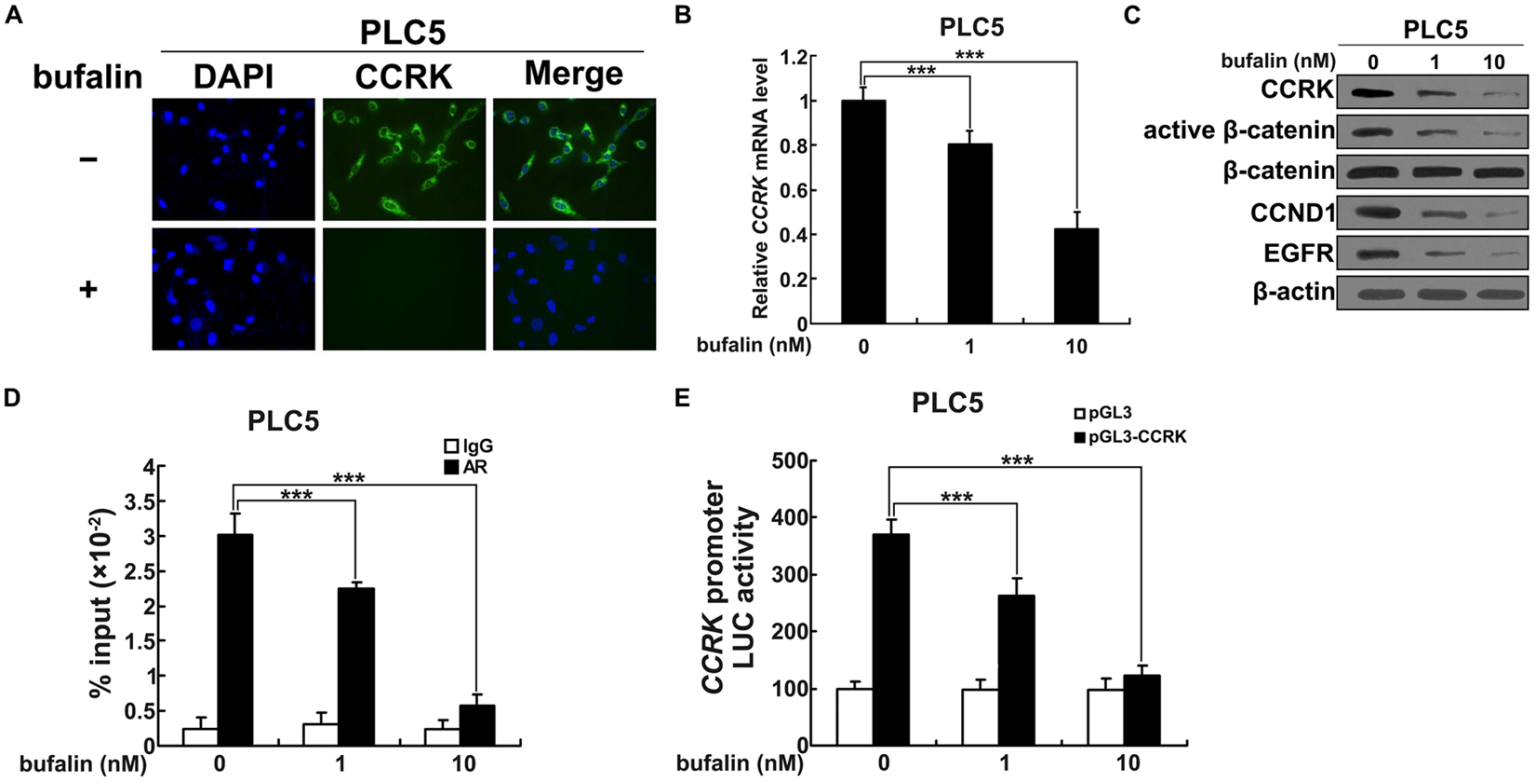
Bufalin reduces CCRK expression by inhibiting the transcription. (**A**) Bufalin decreased CCRK expression in the perinuclear region. Green immunofluorescence staining of CCRK was shown in representative images and nuclei were counterstained with DAPI. (**B**) Bufalin decreased *CCRK* mRNA level in a dose-dependent manner. Various concentrations of bufalin were incubated with PLC5 cells and quantitative RT-PCR was performed to detect *CCRK* transcript levels. (**C**) Bufalin inhibited CCRK expression and inactivated CCRK-induced β-catenin signaling in a dose-dependent manner. Western blot analysis was performed to detect the expression of CCRK, active and total β-catenin, CCND1 and EGFR. β-actin was used as a loading control. (**D**) Bufalin impeded the binding of transcriptional factor to *CCRK* promoter in PLC5 cells. Bufalin or vehicle was incubated with PLC5 cells and anti-AR antibody was used to pulldown antigen-*CCRK* promoter complex for quantitative PCR analysis. IgG antibody was used as a control in ChIP assay. (**E**) Bufalin impaired *CCRK* promoter activity using luciferase reporter assay. *CCRK* promoter constructor and Renilla were transfected into PLC5 cells followed by the treatment with bufalin or vehicle. Luciferase activities related to Renilla control were measured. ***P < 0.001.

Evidences in our previous studies have revealed that CCRK functions as an important oncogene to promote HCC development by governing cell cycle regulation^23^. Mechanistically, CCRK acts as a master mediator to activate β-catenin in driving β-catenin/TCF-dependent hepatocarcinogenesis. We therefore investigated the effect of bufalin on CCRK expression in PLC5 cells. 10 nmol/L of bufalin or vehicle was incubated with PLC5 cells for 48 hours followed by immunofluorescence staining detection. CCRK expressed high level in the perinuclear region of cells with the treatment of vehicle, whereas dramatically decreased when treated with bufalin (Fig. 3A). Quantitative RT-PCR demonstrated that either 1 nmol/L or 10 nmol/L of bufalin significantly reduced the transcript levels of *CCRK* compared to vehicle group (*P* < 0.001; Fig. 3B). Consistently, the expression of CCRK protein was also markedly decreased by the treatment of bufalin compared with vehicle treatment (Fig. 3C). Of note, β-catenin was subsequently inactivated and the expressions of CCND1 and EGFR were downregulated due to bufalin-induced CCRK suppression (Fig. 3C). We then determined the effect of bufalin on transcriptional activity of CCRK to explore the underlying mechanism of bufalin-inhibited CCRK expression. Quantitative ChIP-PCR assay showed that both 1 nmol/L and 10 nmol/L of bufalin significantly abrogated the occupancy on *CCRK* promoter with transcription factors, e.g. androgen receptor (AR)^23^ (Fig. 3D), which inhibited the initiation of *CCRK* transcription. Moreover, luciferase reporter assay showed that bufalin markedly lowered the promoter activity of *CCRK* dose-dependently compared with vehicle (Fig. 3E), which sequentially reduced the efficacy of CCRK transcription. Collectively, these data suggest that bufalin inhibits CCRK expression through the suppression of CCRK transcriptional activity.

### CCRK-mediated β-catenin/TCF signaling is involved in bufalin-induced HCC suppression

**Figure 4:**
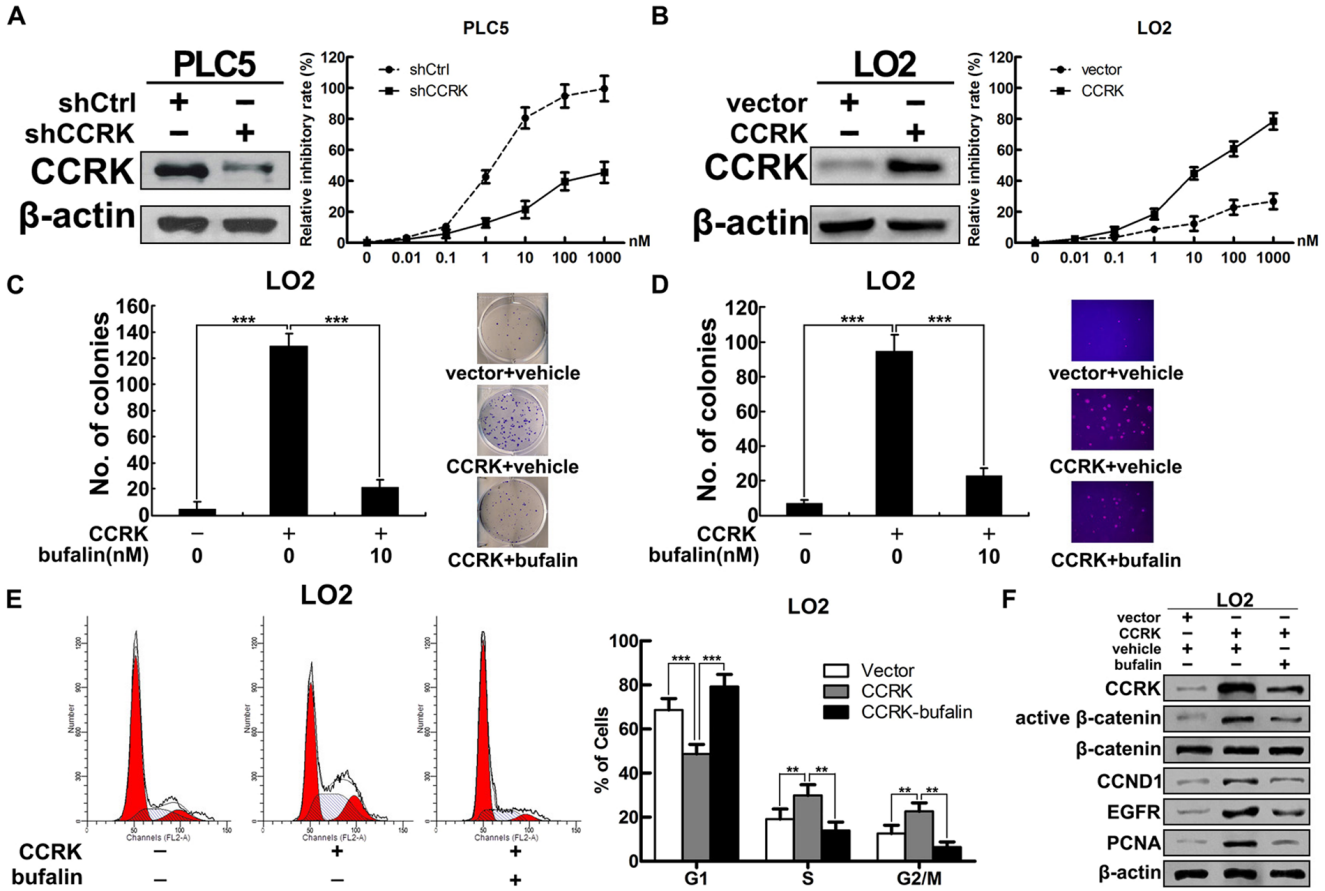
Bufalin blocks HCC cell proliferation and transformation through CCRK-mediated β-catenin/TCF signaling. (**A**) CCRK knockdown attenuated the effect of bufalin on suppressing cell proliferation. Stably CCRK-depriving PLC5 cells or parental cells were treated with indicated concentrations of bufalin or vehicle. Cell growths were detected using CCK-8 assay. Inhibitory rates were plotted as the percentage of viable cells treated with vehicle. (**B**) CCRK expression promoted bufalin-induced cell suppression. Stable CCRK-expressing LO2 cells or parental cells were treated with indicated concentrations of bufalin or vehicle followed by the detection of cell growths in CCK-8 assay. (**C** and **D**) Bufalin abrogated CCRK-induced anchorage-dependent (**C**) or -independent cell growth **(D)** detected by colony formation or soft agar assays, respectively. Representative images of colonies formed are shown. Original magnification ×100. (**E**) Bufalin impeded CCRK-induced G1/S cell cycle progression. (**F**) Bufalin suppressed CCRK-induced β-catenin/TCF signaling activity. Protein expressions of CCRK, active and total β-catenin, CCND1, EGFR and PCNA were detected and β-actin was used as a loading control. **P < 0.01; ***P < 0.001.

To further investigate the role of CCRK in bufalin-induced HCC suppression, we transfected short hairpin RNA against CCRK (shCCRK) into PLC5 cells and constructed stable CCRK-depriving PLC5 cells. Cell growth analysis showed that knockdown of CCRK significantly reduced the effect of bufalin on suppressing cell growth compared with parental cells (shCtrl) at each indicated dosage (Fig. 4A). Noticeably, the concentration of 100 nmol/L almost killed all parental PLC5 cells, but the inhibitory efficacy dramatically decreased to 40% of cell death in CCRK-depriving PLC5 cells. Conversely, in stable CCRK-expressing LO2 cells, ectopic CCRK expression markedly increased the inhibitory effect of bufalin on cell growth dose-dependently (Fig. 4B), demonstrating the importance of CCRK in bufalin-induced cell suppression. To further determine the responsibility of CCRK in bufalin-inhibited cell proliferation and malignant transformation, colony formation and soft agar assay were performed in CCRK-expressing LO2 cells treated with bufalin or vehicle. Enforced CCRK expression in LO2 cells greatly increased the number of colonies in the treatment with vehicle, whereas bufalin significantly counteracted the effect of CCRK on inducing focus formation (Fig. 4C). In accord, CCRK expression in LO2 cells promoted anchorage-independent growth in soft agar, which could be dramatically abrogated by bufalin (Fig. 4D). These data suggested that bufalin effectively inhibited CCRK-induced cell proliferation and malignant transformation. We next evaluated the functional significance of bufalin in CCRK-induced cell cycle progression. Ectopic CCRK expression promoted G1/S phase transition in LO2 cells, while bufalin abrogated CCRK-driven cell cycle progression by arresting cells at G1 phase (Fig. 4E). In accordance with functional analysis, western blot confirmed that CCRK overexpression promoted cell proliferation by activating β-catenin/TCF signaling, which however significantly abrogated by bufalin (Fig. 4F). Taken together, these results strongly support that CCRK-mediated β-catenin/TCF signaling plays a critical role in bufalin-suppressed HCC cell proliferation and transformation.

### Bufalin inhibits hepatocarcinogenesis through CCRK-induced β-catenin/TCF signaling *in vivo*

**Figure 5:**
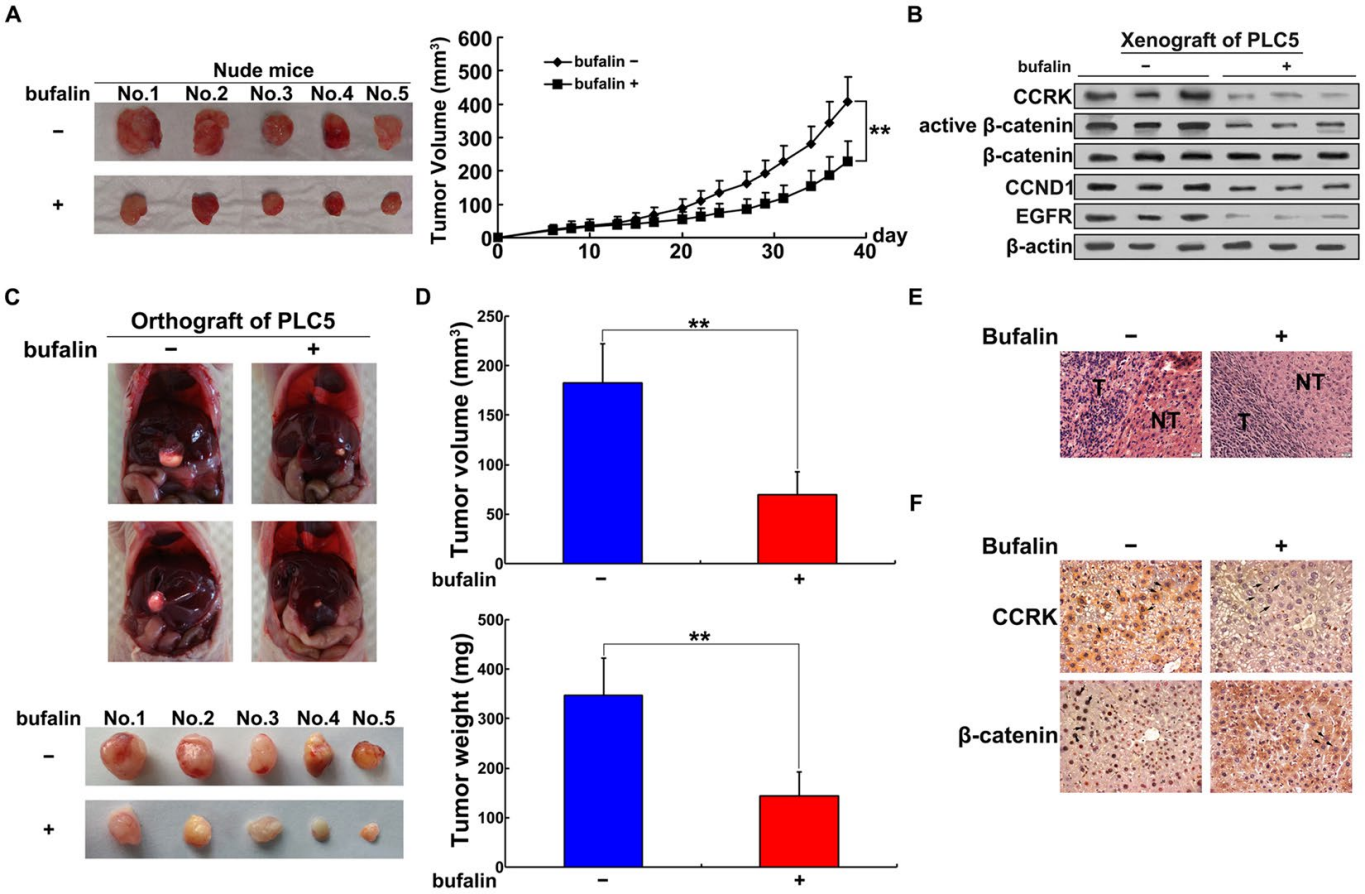
Bufalin inhibits CCRK-driven β-catenin/TCF signaling-dependent tumorigenicity in nude mice. (**A**) Bufalin decreased CCRK-induced tumor growth in xenograft model compared with vehicle treatment. (**B**) Bufalin inhibited CCRK expression and then inactivated CCRK-induced β-catenin signaling. Expression levels of CCRK, active and total β-catenin, CCND1 and EGFR were detected in western blot. β-actin was used as a loading control. (**C**) Bufalin inhibited CCRK-induced intrahepatic neoplasm formation in orthotopic model compared with vehicle treatment. (**D**) Bufalin inhibited CCRK-induced tumor growth in orthotopic model. The tumor volume and weight were measured and compared between two groups treated with bufalin or vehicle. (**E**) Representative images of tumor and nontumor tissues in orthotopic HCC mouse models were shown in hematoxylin and eosin (H&E) staining. Mouse models were treated with bufalin or vehicle. (**F**) Immunohistochemical staining of CCRK and β-catenin in the representative section from orthotopic mouse model. Positive cytoplasmic staining of CCRK and positive nuclear staining of β-catenin were observed in vehicle group, while bufalin treatment decreased the staining strength of CCRK in the cytoplasm and restricted β-catenin in the cytoplasm. **P < 0.01.

We next determined whether bufalin inhibited tumorigenesis through the inactivation of CCRK-regulated signaling by xenograft experiments. The nude mice were randomly divided into two groups (n = 5 per group) and the right dorsal flank of each mice were subcutaneously injected with PLC5 cells. 6 days after cells injection, bufalin or vehicle were individually delivered to mice through tail vein injection three times per week. It can be obviously observed that bufalin significantly attenuated the tumor formation and progression in PLC5 cells inoculated xenograft model within 40 days follow-up (Fig. 5A). Western blot analysis on xenograft tumor tissues further confirmed that the reduced tumorigenicity was associated with the inhibition of CCRK expression and the downstream β-catenin/TCF signaling pathway (Fig. 5B). We then used orthotopic model to verify these *in vivo* findings. Xenograft tumors derived from subcutaneously injected PLC5 cells were harvested and minced into pieces of 1 mm^3^ tissue cubes. Each piece of tumor mince was implanted into the liver of nude mouse and all mice were randomized to classify two groups treated with bufalin or vehicle through the tail vein injection, respectively (n = 5 per group). After 5 weeks, the tumors treated by bufalin or vehicle were excised from livers (Fig. 5C) and their volume and weight were measured. Both the volume and weight of tumors treated by bufalin were significantly decreased compared with those treated by vehicle. The mean volume of bufalin-treated tumors was nearly 3-fold smaller than the ones treated by vehicle, which was in consistent with the change of tumor weight (Fig. 5D). The tumor tissues from bufalin- and vehicle-treated groups were further confirmed by H&E staining (Fig. 5E) and immunohistochemical staining demonstrated expression of CCRK and β-catenin in cancerous lesion tissues (Fig. 5F). Compared with vehicle group, bufalin treatment greatly decreased CCRK expression in the cytoplasm, thus preventing β-catenin entering the nuclei to stimulate cell cycle progression and aberrant proliferation. Altogether, these results confirm that CCRK-regulated β-catenin/TCF signaling pathway is involved in bufalin-induced tumor suppression.

## Discussion

Clinical therapy for human hepatocellular carcinoma, especially in patients with advanced stage, is still a major medical problem worldwide due to the absence of effective chemotherapeutic drugs^29,30^. Bufalin extracted from the traditional Chinese medicine, Chansu, has been well studied to possess potent anticancer activity against a wide spectrum of cancer cells including hepatoma cells^31,32^. However, the precise mechanism of bufalin in inhibiting HCC development and the underlying therapeutic targets are not yet well established. Although a few studies have demonstrated the involvement of apoptosis in the pathological process of bufalin-induced hepatoma suppression^33,34^, it is not fully explained the effect of bufalin on aberrant proliferation of HCC. Related to this, other studies showed that the regulation of cell cycle progression is implicated with the antitumor activity of bufalin in human hepatoma cells such as Huh7, Hep3B and HA22T cells^35,36^. Therefore, it is worthy of further investigation to provide more experimental evidence for the mechanism of bufalin in the treatment of HCC.

In our present study, we found that bufalin effectively induced cell death of PLC5 HCC cells in a dose- and time-dependent manner, while it has minimal cytocidal effect on human immortal hepatocytes LO2 cells. The IC_50_ of bufalin on PLC5 cells hardly inhibited cell growth of LO2 cells, which suggested higher selectivity of bufalin against tumor cells rather than normal hepatocytes. *In vitro* functional analysis further revealed that bufalin dose-dependently abrogated cell proliferation and malignant transformation of PLC5 cells in colony formation and soft agar assay. These results strengthened the potential of bufalin to be exploited as a therapeutic agent in liver cancer treatment^15,37^. Noticeably, we could not find the expression of apoptotic marker active caspase3 and cleaved PARP in the bufalin-treated PLC5 cells, indicating no involvement of apoptosis in PLC5 HCC suppression. In cell cycle analysis, PLC5 cells treated with bufalin were investigated using flow cytometry. Bufalin accumulated cells at G1 phase dose-dependently without increasing the typical sub-G1 group for programmed cell death, suggesting the mechanism of G1 phase arrest and non-apoptotic regulation involved in antitumor activity of bufalin. Interestingly, bufalin has also been shown to arrest cell cycle at G2/M phase and induce cell death by autophagy instead of apoptosis in hepatoma cells^35^. The similar findings were also demonstrated in the bufalin analogue arenobufagin, which showed the antitumor activity by causing G2 phase arrest in cell cycle through the regulation of ATM/ATR signaling pathway^38^.

CCRK is a mammalian cyclin-dependent kinase (CDK) family member that plays an indispensable role in cell growth^39,40^. Dysregulation of CCRK activity in cancer cells provokes tumor-associated cell cycle defects to induce unscheduled proliferation^41^. In our previous studies, we revealed that CCRK has the oncogenic property to promote cell proliferation and malignant transformation in HCC cells and to induce tumorigenicity in mice model^23^. In this study, we found that bufalin could eliminate the level of *CCRK* transcript and protein expression in PLC5 HCC cells in a dose-dependent manner. The performance of promoter binding (ChIP) and luciferase reporter assays demonstrated the dual role of bufalin in the regulation of *CCRK* transcriptional activity. Bufalin abrogated the transcriptional activity of *CCRK* not only by abating the combination of *CCRK* promoter with transcription factors, but also by inhibiting the activation of *CCRK* promoter. For instance, AR acted as a transcription factor to upregulate *CCRK* transcription by binding to its promoter^23^, while bufalin significantly inhibited the occupancy of AR on *CCRK* promoter, thus suppressing hepatocellular neoplastic development. These data provide compelling evidence that bufalin repressed CCRK expression through the modulation of transcriptional activity. Of note, bufalin has also been reported to inhibit AR activation by suppressing the steroid receptor co-activator SRC-3 without binding to the receptor itself^37^, suggesting the key role of AR in bufalin-induced HCC suppression. Based on these and our findings, it is thus tempting to propose that AR inactivation by bufalin is an alternative mechanism for bufalin-regulated *CCRK* transcription in HCC. However, further molecular evidence is needed to validate this hypothesis.

The carcinogenic β-catenin/TCF signaling is frequently activated in human HCCs^18^. Compared with genetic defects of *CTNNB1*, the occurrence of abnormal accumulation and activation of β-catenin is more frequent in aberrant β-catenin/TCF signaling in HCCs^42,43^. Recent findings demonstrated that endogenous expression of CCRK protein in hepatoma cells activates β-catenin/TCF signaling and in turn prompts the expression of downstream targets, such as CCND1 and EGFR^44–46^. CCND1 regulates cell transition from G1 to S phase by combining with cyclin-dependent kinase in G1 phase, thus governing the process of cell proliferation^47^. In our present study, we found that bufalin could eliminate the level of *CCRK* transcript and protein expression in PLC5 cells in a dose-dependent manner. The inhibition of CCRK induced by bufalin reduced the activity of β-catenin/TCF signaling and subsequently suppressed the expression of CCND1 and EGFR, which impeded cell proliferation of PLC5 HCC cells. Importantly, CCND1 downregulation could cause G1/S phase arrest, providing the evidence that antitumor activity of bufalin is connected with cell cycle regulation.

The functional link between bufalin and CCRK was further demonstrated *in vitro* and *in vivo* experiments. Silencing of CCRK expression in PLC5 cells could decreased the inhibitory efficacy of bufalin on cell growth, while in the reciprocal experiment, ectopic CCRK expression in LO2 cells could significantly increase the selectivity of bufalin to induce cell death of normal hepatocytes in a dose-dependent manner. Moreover, CCRK expression promoted cell proliferation and malignant transformation, which could be dramatically counteracted by bufalin treatment. In parallel, ectopic CCRK expression in LO2 cells prompted cells from G1 phase into S phase in cell cycle progression, which was abrogated by bufalin with G1 phase arrest. *In vivo* study, bufalin significantly reduced tumorigenicity in both the xenograft and orthotopic models, accompanied with the inhibition of CCRK expression, inactivation of β-catenin and decrease of CCND1 and EGFR expression, which is related to cell cycle arrest and suppression of cell proliferation.

In conclusion, we demonstrated for the first time that CCRK is the major therapeutic target for bufalin to suppress human HCCs. In mechanism, bufalin inhibits *CCRK* transcription and its protein expression, thus inactivates β-catenin signaling and decreasing the downstream factors CCND1 and EGFR. The blockage of CCRK/β-catenin signaling is associated with the G1 phase arrest in cell cycle, leading to hepatic tumor suppression. Our results thus provide new evidence to support the therapeutic usage of bufalin on HCC treatment and are helpful to develop the analogues for this kind of new therapeutic strategy.

## Methods

### Cells, reagents and expression vectors

HCC cell line PLC5 and normal immortalized liver cell line LO2 were maintained in the high-glucose DMEM media (Gibco) supplemented with 10% fetal bovine serum (FBS; Hyclone). The cells were cultured at 37 °C in 5% CO_2_-containing humidified incubator. Bufalin was purchased from Sigma and prepared for the store solution by dissolved in DMSO. In the experiments, store solution of bufalin was diluted with culture media into indicated concentration and incubated with cells. CCRK-expressing vector and short hairpin RNA vector targeting CCRK (shCCRK) were kindly provided by Marie Lin in Chinese University of Hong Kong. Vectors were transfected into cells with lipofactamine 2000 (Invitrogen) according to the manufacturer’s instructions. For the construction of stable CCRK-depriving PLC5 and stable CCRK-expressing LO2 cells, transfected cells were transferred into selective antibiotics and cultured until the growth of antibiotics-resistant colonies.

### Cell viability assay

Cell viability was detected using CCK-8 kit (AbMole). In brief, cells (2 × 10^3^ /well) were seeded in the 96-well plate covered with 10% FBS-containing DMEM media and cultured overnight. Various concentrations of bufalin were incubated with cells for 48 hours or 10 nmol/L bufalin was incubated with cells for consecutive 5 days. Cells were then treated with CCK-8 reagent for 1 hour in the incubator. Optical density was measured using microplate reader (Thermo Scientific) in triplicate and the mean value of absorbance was referred to the quantity of viable cells. IC_50_ of bufalin in PLC5 cells for 48 hours was calculated using GraphPad Prism 5 (GraphPad Software, Inc.).

### Colony formation assay

PLC5 or stably transfected LO2 cells (10^3^/well) were seeded in 6-well plates and cultured overnight to adhere to the plate. Cells were then treated with bufalin at different concentration or vehicle alone for 3 days. After treatment cells were cultured in bufalin-free media for 2 weeks. The colonies formed were stained with 0.2% crystal violet and photographed. Numbers of colonies were counted under the microscope. Data were obtained from 3 independent experiments.

### Soft agar assay

6-well plates were covered with a layer of 0.6% agar in DMEM media in advance. PLC5 or stably transfected LO2 cells (2 × 10^3^/well) were mixed with 0.3% agar in bufalin-containing DMEM media and seeded in triplicate onto the 6-well plate. The mixture of cells and agars was covered with bufalin-free 10% FBS-containing DMEM media and cultured for 3 weeks. The bufalin-resistance colonies were formed in the agar and stained with 0.2% crystal violet. Numbers of colonies were counted under the microscope. Data were obtained from 3 independent experiments.

### Cell cycle analysis

Cell cycle analysis was performed using propidium iodide staining (PI; Sigma) in flow cytometry. PLC5 or stably transfected LO2 cells were treated with bufalin or vehicle for 24 hours. Cells were washed with PBS and fixed in ice-cold 70% ethanol for 30 minutes followed by the denaturation in 4N hydrochloric acid for 20 minutes at room temperature. The cells were then stained with 50 μg/ml PI solution containing 380 mM Sodium Citrate and 10 μg/ml RNase A for 1 hour. Cellular DNA content was measured using FACS Calibur Flow Cytometer (BD Bioscience) and analyzed by WinMDI2.9 software. Data were obtained from 3 independent experiments.

### Immunofluorescence staining

PLC5 cells were plated on glass coverslip and cultured overnight to 30% confluence. Cells were then treated with bufalin or vehicle for 24 hours followed by fixation with 3% paraformaldehyde and permeation with 0.1% Triton X-100. Nonspecific binding sites were blocked with 1% BSA for 30 minutes. The cells were incubated with primary mouse anti-human CCRK antibody (Sigma) or rabbit anti-human β-catenin antibody (Abcam) for 1 hour, and then incubated with FITC-conjugated goat-anti-mouse antibody (Invitrogen) or rhodamine-conjugated goat-anti-rabbit antibody (Invitrogen) for 30 minutes. Nuclei were counterstained by DAPI (Invitrogen) and Images were captured using confocal microscope (Olympus).

### Reverse transcription and quantitative PCR

PLC5 cells were treated with bufalin or vehicle for 24 hours. Total RNA was isolated from cells using Trizol reagent (Invitrogen) and quantified using Nano-drop (Thermo Scientific). 1 μg RNA was reversely transcribed to cDNA using Reverse Transcription Master Kit (Invitrogen) according to the manufacturer’s instructions. cDNA was aliquoted and amplified in triplicate to quantitate *CCRK, CTNNB1, CCND1 and EGFR* transcript levels using Power SYBR Green PCR Master Mix (TaKaRa) in 7500 Fast-Real-Time PCR system (Applied Biosystems). *GAPDH* was used as an internal control.

### Immunoblotting analysis

Protein lysates were extracted from cells using RIPA lysis buffer containing protease inhibitor cocktail (Roche). Protein concentration was quantified by Bradford method (Bio-Rad laboratories) according to the manufacturer’s instructions. Aliquot amount of protein lysates were resolved in 10% SDS-polyacrylamide gel, separated by electrophoresis and electro-transferred into equilibrated nitrocellulose membrane (Bio-Rad Laboratories). Membranes were incubated with Primary antibodies at 4 °C overnight followed by HRP-conjugated secondary antibodies for 1 hour at room temperature. Antigen-antibodies complexes in the membranes were detected using the western blotting Chemiluminescence Luminol Reagent (Amersham). The corresponding bands were exposed on X-ray films for less than 2 minutes and scanned with 300 dpi of resolution using CanoScan LiDE 120 color image scanner (Canon). Primary antibodies used in this study include rabbit anti-CCRK (Abcam, Cat#ab227077; 1:1000), mouse anti-active β-catenin (Millipore, Cat#05-665; 1:2000), rabbit anti-β-catenin (Cell signaling, Cat#9562; 1:1000), rabbit anti-CCND1 (Invitrogen, Cat#PA5-12255; 1:1000), mouse anti-EGFR (BD laboratories, Cat#ABIN126775; 1:3000), mouse anti-PCNA (Abcam, Cat#ab201673; 1:2000), rabbit anti-caspase3 (Abcam, Cat#ab115183; 1:1000), rabbit anti-active caspase3 (Abcam, Cat#ab49822; 1:1000), rabbit anti-PARP (Abcam, Cat#ab227244; 1:2000), rabbit anti-cleaved PARP (Abcam, Cat#ab4830; 1:1000), rabbit anti-CDC25A (cell signaling, Cat#ab3652; 1:1000), rabbit anti-CDK6 (Abcam, Cat#ab151247; 1:1000), rabbit anti-CDKN1B (Abcam, Cat#ab215434; 1:1000) and mouse anti-β-actin (Sigma, Cat#A1978; 1:15000). HRP-conjugated Secondary antibodies include goat anti-rabbit (Santa cruz, Cat#sc2004; 1:5000) and goat anti-mouse (Santa cruz, Cat#sc2005; 1:5000).

### Chromatin Immunoprecipitation coupled with quantitative PCR

PLC5 cells treated with bufalin or vehicle for 24 hours were crosslinked with 1% formaldehyde for 10 minutes at room temperature. The cells were then washed by PBS, lysed by lysis buffer, and the chromatin in the lysates was fragmented into 100~500 bp by sonication. The protein-DNA complexes were pulled down by anti-AR antibody (Cell signaling) or anti-IgG antibody (Sigma) binding to Dynal magnetic beads (Invitrogen). The immunoprecipitated (IP) and input complexes were then washed and reversely crosslinked to release protein-bound DNA. Aliquot amount of IP and diluted input DNA were used for quantitative PCR analysis based on Power SYBR Green detection (Applied Biosystems) as previously described.

### Luciferase reporter assay

*CCRK* promoter luciferase reporter was constructed as described in our previous study^23^. PLC5 cells were transfected with *CCRK* promoter constructors and Renilla luciferase reporters for 48 hours followed by the treatment with bufalin or vehicle for 24 hours. Cells were then collected and assayed using the Dual Luciferase Reporter Assay System (Promega) in GloMax 96 microplate luminometer (Promega). All experiments were done in triplicate and for 2 independent experiments.

### Xenograft mouse model

Studies using female athymic nude mice (4 to 6 weeks old) were approved by the animal experimentation ethics committee of Shuguang Hospital, Shanghai University of Traditional Chinese Medicine. The experimental procedures in our studies were confirmed with the National Institute of Health Guidelines for the Care and Use of Laboratory Animals. 5 × 10^5^ PLC5 cells were subcutaneously injected into the right flank of nude mice and tumor appeared visible in six days. Nude mice were then treated with bufalin or vehicle three times per week (n = 5 per group). Tumor size was measured using a caliper every other day, and tumor volume was calculated according to the formula of 0.5 × length × width × width. The mice were sacrificed at 5~6 weeks and tumors were stored for protein analysis.

### Orthotopic mouse model

5 × 10^5^ PLC5 cells were subcutaneously injected into the right flank of athymic nude mice to construct xenograft mouse model. Mice were sacrificed 4 weeks post injection and tumors were harvested to cut into several pieces of 1 mm^3^ cube. Each tumor cube was implanted into the left liver lobe, and then nude mice were treated with bufalin or vehicle three times per week 6 days post implantation (n = 5 per group). After 6 weeks, mice were sacrificed and tumors were excised. Tumor volume and weight were measured.

### Immunohistochemistry

Freshly cancerous lesion liver tissues isolated from orthotopic mouse models were fixed in 10% formalin, dehydrated, embedded in paraffin and sliced. The sections were then deparaffinized in xylenes and rehydrated by a graded series of alcohols, followed by antigen retrieval and blockage of endogenous peroxidase activity by 3% hydrogen peroxide in methanol. Tissue sections were incubated with primary antibodies against CCRK (1:25, Abcam) and β-catenin (1:50, Abcam) for 2 hour. Followed chromogen development was automatically accomplished with EnVision + polymer system (Dako). Images were obtained by Nikon microscope and the staining intensity was categorized into 0 (none), 1 (weak), 2 (intermediates) and 3 (strong) in terms of proportion of positive tumor cells.

### Statistics

Unless otherwise mentioned, all data were presented as mean + standard deviation of three independent experiments. The independent Student *t* test was performed to examine the difference between 2 groups using GraphPad Prism 5 (GraphPad software). A two-tailed *P* value of less than 0.05 was considered statistically significant.

### Data Availability

All data generated or analysed during this study are included in this published article.
